# Combined treatment with paraffin, manual therapy, pegboard and splinting in a patient with post-traumatic stiff hand

**DOI:** 10.1186/s40945-016-0028-y

**Published:** 2016-11-29

**Authors:** Eva Santacreu Santacreu, Núria Villanueva Cabezas, Asunción Bosch Graupera

**Affiliations:** Rehabilitation Service, Area of Traumatology, Hospital Universitari Vall d’Hebron, Pg Vall d’Hebron 119-129, Barcelona, 08035 Spain

**Keywords:** Stiff hand, Manual therapy, Pegboard, Splints, Paraffin

## Abstract

**Background:**

The stiff hand is a still common, severe complication of hand injuries.

**Case presentation:**

We report here the case of a 56 year-old woman, professional goldsmith, who suffered a distal radius fracture of her right hand. The patient was treated with surgery followed by four weeks of immobilization, and developed a stiff hand. Physical examination showed mild inflammatory signs, pain and a major limitation in the extension and supination of the wrist, and in the mobility of the II, III, IV and V metacarpophalangeal (-5° and 32° of average passive extension and flexion, respectively) and interphalangeal (-35° and 73° of average passive extension and flexion, respectively) joints. There was a lack of slip of the flexor tendons. The diagnosis of complex regional pain syndrome was considered although it could not be definitely established. After five months of adverse evolution the patient was referred to our center where a combined intervention with paraffin, manual therapy, prolonged active and passive stretch on a pegboard, and splinting was applied. After initiation of this therapy, a marked change in the evolution of the pain, the mobility and functionality of the hand was observed. At the end of the rehabilitation program the patient was able to fully resume her job.

**Conclusion:**

The present case illustrates the need of intensive treatment for post-traumatic hand stiffness, and describes, as an original contribution, a combined intervention therapy including paraffin, manual therapy, pegboard and splinting.

## Background

The stiff hand is a still common complication of hand injuries. Scar tissue can affect the skin, fascia, tendons, muscles, ligaments and joint capsules, restricting movement amplitude and limiting function [1]. We use the term stiff joint to refer to a restriction of the range of motion and a stiff end feel [2]. The best way to avoid the functional consequences of a stiff hand is to prevent its development. Available knowledge of the phases of tissue repair and healing supports an early initiation of rehabilitation in these patients [3]. Currently, there are no clear and universally accepted recommendations for the treatment of the stiff hand. In addition to the severity and type of trauma, repetitive surgery, the occurrence of complications as infections or persistent edema, delayed or inadequate treatment [4] is an important determinant of the occurrence of significant movement restrictions that hinder the proper function of the hand and deteriorate the quality of life of patients that have suffered a hand traumatism.

There is evidence that prolonged, low intensity stretching may allow beneficial scar elongation and remodeling. Arem and Madden showed in the seventies, based on studies in rats, that the orientation of collagen fibers in an immature scar could be modified by the application of prolonged tension [5]. Connective tissue responds to external stress forces in two ways, with elasticity (returning to its previous position after cessation of tension), and plasticity (permanent deformation). Plastic deformation can be induced with prolonged stimuli of low intensity [2, 6]. Light et al. showed in 1984, a group of elderly subjects with knee flexion contracture that mobilization with low load and sustained in time was more effective than brief mobilization with high load [7].

A convenient way to apply prolonged, low intensity stretching to the stiff hand is the use of a workbench pegboard. However, although the use of pegboard in the treatment of the stiff hand was described several decades ago, there is a lack of reports describing this technique and and analyzing its efficacy. We present a case describing a strategy to treat the stiff hand based on the application of a combined therapy including the use of the pegboard in addition to applied paraffin, manual therapy and splinting.

## Case presentation

A 56 years old female, goldsmith by profession, suffered a distal radial fracture of the right hand that was treated with surgery, involving the implant of plates and screws, followed by forearm immobilization with a cast for four weeks. Once the cast was removed, physiotherapy was started with application of intense and painful manual mobilization and exercises against resistance, as described by the patient. The function of the hand was not recovered; on the contrary, the patient experienced a progressive limitation of active and, later, passive movement, as well as persistent signs of inflammation. After five months, the patient was referred to our center.

At physical examination the hand was painful, and showed mild swelling with partial disappearance of the folds of the hand and fingers, and with redness of the metacarpophalangeal and interphalangeal joints. There was a reduction of both active and passive movement of the wrist, the metacarpophalangeal, and the proximal and distal interphalangeal joints as measured by using a two arms goniometer and a fingers goniometer (Saehan, Korea) and the Kapandji index. This index of thumb opposition mobility ranges between 1 and 10, 1 indicating minimal mobility (the thumb tip reaches the lateral side of the second phalanx of the 2nd finger) and 10 indicating maximal mobility (the thumb tip reaches the distal palmar crease at the base of the 5th finger) [8] (Table 1). There was stiffness of the wrist in flexion, and of the metacarpophalangeal joint in extension, due to retraction of the collateral ligaments. There was also stiffness of the proximal interphalangeal joint in flexion, due to retraction of the palmar plate, and stiffness of distal interphalangeal joint in flexion. The thumb was in slight adduction, with stiffness of the joint and contracture of the adductor and first dorsal interosseous muscle. Joints mobilization showed a firm end feel at the extremes of the restricted movement suggestive of established stiffness.

**Table 1:** Active (A) and passive (P) ranges of movement of the wrist and fingers joints at baseline, at nine weeks and at nine months (end of the treatment)

	Baseline	At nine weeks	At nine months
Wrist extension/flexion	A −5/15	A 20/50	A 45/70
	P −5/20	P 30/50	P 50/70
Wirst pronation/supination	A 80/0	A 90/55	A 90/80
	P 80/0	P 90/60	P 90/80
Wrist radial/ulnar deviation	A 5/5	A 25/30	A 25/30
	P 5/5	P 30/35	P 30/35
Extension/flexion of trapeziummetacarpal joint of the thumb	A 10/15	A 30/25	A 30/25
	P 10/20	P 30/25	P 30/25
Extension/Flexion of the metacarpophalangeal joint of the thumb	A −5/30	A −5/45	A 0/45
	P −5/40	P −5 /45	P 0/45
Extension/flexion interphalangeal joint of the thumb	A 0/50	A 0/75	A 0/90
	P 0/60	P 0/75	P 0/90
Average extension/flexion of the metacarpophalangeal joints of II, III, IV and V fingers	A −5/22.5	A 0/44	A 0/75
	P −5/32.5	P 0/52.3	P 0/75
Average extension/flexion of the proximal interphalangeal joints of II, III, IV and V fingers	A −32.5/72.5	A −22.5/95	A −15/100
	P −35/73	P −25/90	P −10/100
Average extension/flexion distal of the interphalangeal joints of II, III, IV and V fingers	A −4/34	A 0/72.5	A 0/90
	P 0/52.5	P 0/72.5	P 0/90
Average distance from finger tips to palmar crease in II- V fingers	8 cm	4 cm	0 cm
Kapandji	Finger tip of IV finger	Distal palmar crease of V finger base	End palmar crease of V finger

Kapandji (thumb mobility index)

The patient did not show any area of hypoesthesia or allodynia. Hypoesthesia was assessed by touching the skin with a small cotton swab and allodynia was examined by touching the skin with the tip of a needle in different areas of the hand and the forearm and asking the patient to tell whether she could notice it or feel pain according to a verbal numeric scale (VNS), with point 0 representing no pain and point 10 the worst possible pain.

The intensity of pain was 4/10.

At the tendons level, a lack of sliding was observed in the flexor tendons. A marked difference in the easy of distal joint motion was noticed depending on whether the proximal joint was in flexion or in extension. A palmar scar was present in the wrist attached to deeper structures and restricting tendon slip.

Force assessment was not performed due to range of motion restrictions.

The Disabilities of the Arm, Shoulder and Hand (DASH) questionnaire was used to evaluate the functionality of the hand. The DASH is scored 0-100 in two components: a disability/symptoms section (30 items) and an optional sport/music or work section (4 items each). A higher score indicates greater disability. The initial score was 89/100 and 100/100 for the disability/symptoms section and the optional section, respectively.

The diagnosis of complex regional pain syndrome (CRPS) was considered, as the patient fulfilled the Budapest criteria published by the IASP in 2007 (pain, swelling, peri-articular skin redness and progressive stiffness) [9]. According to the Kozin’s criteria, the diagnosis of CRPS was “probable” in our patient [10]. However, pain can spontaneously decrease with time in CRPS, and mild osteoporosis may not be detected by conventional Xray examination [11]. Thus, although the diagnosis of CRPS could not be definitely established in in this patient, we considered it likely.

Our main goal was to restore the range of motion by submitting scar tissues to adequate tension forces to elongate them and allow movement. For this purpose we used four simple, non-aggressive techniques: paraffin, manual therapy, work in pegboard and splinting.

During the physiotherapy sessions, manual drainage techniques and compression bandaging were applied to reduce swelling and minimize the generation of scar tissue, and the patient was taught to maintain the arm in an elevated position and to perform gentle active movements.

Therapy was applied at hospital in daily sessions of up to three hours, depending on the tolerance of the patient, during 9 weeks. After observing the good response to this treatment (see Table 1), it was prolonged until the sixth month of on an outpatient basis. During three subsequent months three treatment sessions per week were performed.

**Fig. 1:**
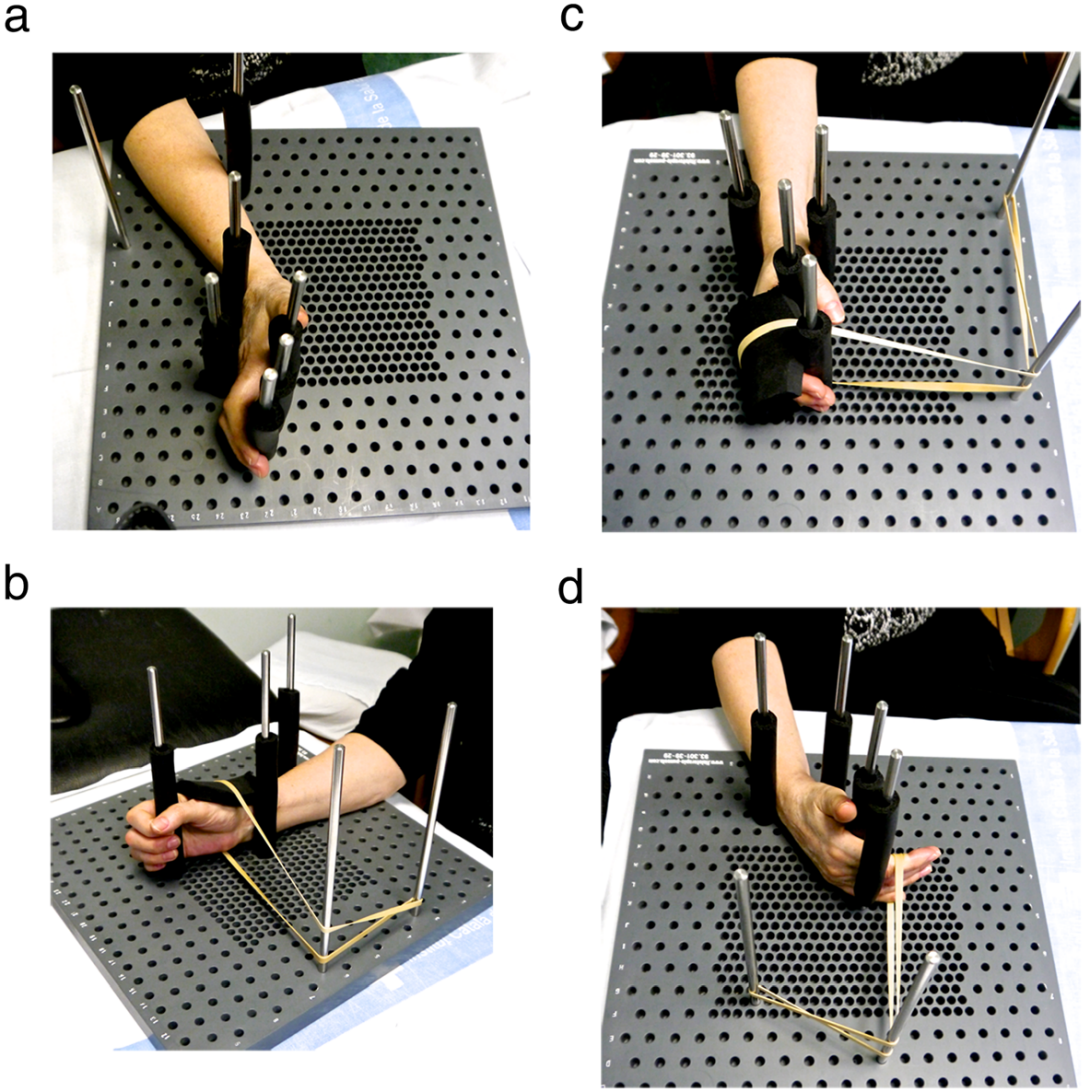
Pegboard work. **a** Sliding of flexors tendons of the hand (zones 4/5). **b** Anterior translation of the first carpal row of the wrist to improve wrist extension. **c** Anterior translation of the first phalanx to improve metacarpophalangeal joint flexion. **d** Posture to improve the extension of the proximal interphalangeal joint

- Paraffin was applied for 20 min prior to manual therapy or together with pegboard work [12].
- Manual Therapy. Mobilization of all joints with restricted movement was performed over 45 min according to the Kaltenborn technique [13]:
  – Mobilization of superior and inferior radioulnar joints by applying dorsal and palmar sliding movements of the radius on ulna (to increase supination and pronation of the forearm);.
  – Dorsal and palmar sliding mobilization of carpus on radius and of capitate on lunate, and mobilization of scaphoid, pisiform and triquetrum (to gain wrist extension and flexion);.
  – Radial and ulnar sliding mobilization of carpus respect on radius (to gain wrist abduction and adduction);
  – Dorsal and palmar sliding mobilization of each metacarpophalangeal and interphalangeal joint (to gain extension and flexion);
  – Radial and ulnar , and dorsal and palmar sliding movements of first metacarpus (to gain flexion-extension and abduction-adduction r.o.m., respectively, of the thumb carpometacarpal joint;
  – To improve thumb mobility, relieving massage was performed as coadjutant therapy in the first dorsal interosseous and adductor pollicis.
    The remaining time was spent in pegboard work and in the preparation and progressive adjustment of splints.
- Pegboard. Pegboard work (Fig. 1) was used as a mechanotherapy tool in order to shape the joint and tendon adhesions by active sliding tendon postures and passive joint postures.

We used a commercially available pegboard consisting of a 40 cm by 40 cm PVC board with 35 perforations of 8 mm of diameter separated by 2 cm (Atase, Barcelona, Spain). Work was performed with the aid of stainless steel pegs and elastic bands.

One tendon posture and three joint postures were alternated during pegboard work. Each posture was maintained for 20 min. In all four postures, hand and forearm were placed on the pegboard on their ulnar sides.

To stretch the flexor tendons at the wrist (zones 4/5), the first posture used was that of maximum tension of the flexor tendons (outstretched hand). Then, a first peg was placed in the back of the wrist to be used as fulcrum of motion. Two further pegs were placed on the anterior and the posterior surfaces of the forearm, to mark the points of start of stress and of maximal painless stress respectively. The patient was asked to actively move her forearm between the two pegs. As tendon sliding improved, the dorsal peg was displaced to increase tendon tension (Fig. 1a).

The three joint postures were chosen to improve the articular motion that was most restricted. The first posture was used to improve the extension of the wrist. The wrist was placed in maximal painless extension and was fixed in this position with two pegs. An elastic band was passed at the level of the first carpal row. In order to move it forward and elongate the scar tissue in the anterior face of the articular capsule of the wrist. This was intended to increase wrist extension without angular movements potentially causing joint irritation. As the articulation yielded, we tighten the elastic band without causing pain (Fig. 1b).

A second posture was used to improve the elasticity of collateral ligaments and metacarpophalangeal joint flexion. In this posture an anterior translation of the first phalanx was achieved by placing an elastic band at the base of the first phalanx while stabilizing the metacarpophalangeal and wrist joints with pegs (Fig. 1c).

The third posture was aimed to improve the extension of the proximal interphalangeal joint by increasing the elasticity of the anterior ligament. With the aid of pegs the wrist was stabilized in partial extension, and the metacarpophalangeal in relative flexion, to relax the flexor musculoskeletal apparatus. An elastic band was placed at the level of the second phalanx and was progressively tighten to bring the second phalanx backwards (Fig. 1d).

d) Splinting. In the first session, two static splints were made with thermoplastic material. Later the splints were modified as the joint range of motion increased. The patient used these splints throughout the nine months of treatment.

A first splint was made to maintain the metacarpophalangeal joints flexed, while the interphalangeals and the wrist were free and the prehensile function was not restricted. This splint was well tolerated and eventually used the whole day. At week nine, the patient resumed her work, and used the splint only outside working hours.

The second splint was made for the night with slow and progressive wrist extension, flexion of the metacarpophalangeal joints, and extension of proximal and distal interphalangeal joints.

At eight weeks, the patient showed an increase in joint range of motion and freedom of movement, and an additional period of 30 min was dedicated to activities aimed to improve skill, coordination proprioception and strength, as well as to prepare a program of exercises to be performed at home. Home work included: exercises with elastic bands (Theraband), passive automobilizations, and different exercises to improve dexterity and manipulative skills in relation with her profession as goldsmith.

After nine weeks of treatment, at hospital discharge, a clear change in the clinical evolution, with a progressive recovery of the mobility of the hand, was registered.

Goniometry demonstrated an increase in passive and active movement in all rigid joints (Table 1).

There was also a reduction of inflammatory signs, and pain virtually disappeared (VNR=1).

The DASH score for the severity of symptoms and disability was 54/100 and the score of the optional section was 81/100.

During the six month and three weeks period of outpatient rehabilitation the recovery of mobility, dexterity, strength and usefulness of the hand continued to improve.

The improvement in hand mobility was associated with an increase in strength. We evaluated of strength with the aid of a hydraulic grip dynamometer at final discharge (Sahean, Korea). Digitopalmar strength was 20/50 at 9 weeks and 36/50 at 9 months.

At the end of the rehabilitation program the patient was able to fully resume her work as a goldsmith.

## Conclusions

Improper initial treatment after hand trauma, inadequate management of inflammatory signs, especially pain and edema, a too aggressive mobilization, and a late start of treatment favoring the development of stiffness, are all causes of treatment failure. At present, the stiff hand is not an infrequent complication of even simple hand traumatism. In our patient, the development of a stiff hand could have been favored by a too long period of immobilization, the initial treatment with too intense mobilization and early resistance training, or by the presence of complex regional pain syndrome, a less frequent syndrome more difficult to diagnose.

The present case illustrates the beneficial effects of the combination of paraffin, manual therapy, pegboard work and splinting in the treatment of the post-traumatic stiff hand. The initiation of combination therapy was accompanied by a clear change in the evolution with gradual reduction of stiffness. Functional recovery was satisfactory, allowing the patient to resume her work as a goldsmith.

After an injury, connective tissue repair takes place following inflammatory, proliferative and organizational phases which need to be considered to establish an adequate plan of physiotherapy [14, 15]. When the patient initiated the treatment at our center, tissue repair was expected to be in the phase in which collagen fibers are getting organized according to internal and external mechanical stimuli acting on the tissue [15].

According to Kaltenborn [13] manual therapy allows to stretch the shortened tissue increasing the range of motion. However, other authors consider that the use of manual therapy as a tool for the modification of connective tissue requires further investigation [6, 16]. We complemented this treatment with pegboard work and splinting.

Although pegboard work has been used in the treatment of the stiff hand for decades [2, 17], there is scant information on this therapy in the literature. There is a lack of information on the role of pegboard work in the rehabilitation of the stiff hand, the postures to be applied, or the optimal duration of sessions. The use of pegboard work in the treatment of joint stiffness is based on evidence from pre-clinical and clinical studies showing that the application of sustained, low intensity tension to scars tissue may reorientate collagen fibers and result in permanent elongation [2, 5, 7].

We use routinely the pegboard for treating rigidities with application times of at least 20 min. The choice at this duration is based on previous studies showing that it allows elongation of the connective tissue in the supraspinose ligament [18].

In contrast with pegboard, splinting is widely used in the treatment of the stiff hand. Michlovitz recommended the use of splints together with passive mobilization to treat stiffness [19]. Flowers et al., in a study on stiffness of the proximal interphalangical joint of the hand treated with costs, observed that the increase attained in passive range of motion was directly proportional to the length of time during which the joint was held in end of motion. [20]. The optimal duration of the use of splinters may vary according to the clinical condition. A clinical trial involving 43 patients showed a positive effect of the use on splints during 6–12 h per day in patients with post-traumatic hand stiffness [21], while Schultz-Johnson suggests that in patients with established stiffness and hard end of movement splints should be used during the whole day [22].

Limb elevation usually does not resolve chronic swelling. In our case, swelling was reduced after simultaneous application of manual drainage techniques, compressive measures and limb elevation. We think that this may reflect in part the persistent inflammation due to the initial treatment or CRPS.

As a general rule, the use of paraffin is contraindicated in the presence of edema and acute inflammatory signs. Although it has been proposed that paraffin may be useful in the treatment of stiffness [12] even in the presence of mild, chronic inflammation, and despite the good evolution of our patient, we cannot recommend its rutinary use in combination with mobilization, pegboard and splinting in the treatment of stiffness.

In the present case, the combination of paraffin, manual therapy, pegboard work and splinting interrupted the adverse clinical evolution of a patient with post-traumatic stiff hand who had previously received manual mobilization and exercises against resistance, restored the functionality of the hand. Based on this observation, we suggest that combined therapy should be considered in patients with stiff hand.

The described treatment was intensive and prolonged, but we think that it use was justified based on the results obtained. We acknowledge that the cost associated with this treatment may represent a limitation regarding its replication in many clinical settings. However, we are studying ways to make this approach less costly. In this regard, we think that self-administration of part of the treatments at home, including the pegboard exercises, should be possible in many patients. This would require a well defined initial training and scheduled visits for the solution of doubts and adjustments of treatment.

Although recent advances in the cellular biology of the scarring process in stiff joints could eventually lead to a better prevention of joint stiffness [23], improving the efficacy of available treatments is much needed at this moment. Further research on the efficacy of combination therapy with or without pegboard, or with a different planning, including frequency and administration modality, will be necessary to define the best approach to the management of the post-traumatic stiff hand.
